# Temperature-Mediated Structure–Functionality Changes in Soybean Meal Protein via Extrusion

**DOI:** 10.3390/foods15162866

**Published:** 2026-08-17

**Authors:** Rong Ma, Xiqin Pan, Yuhan Zhuang, Yifei Han, Shanshan Li, Zhengfeng Fang, Mingquan Xia, Liang Hu, Hong Chen

**Affiliations:** College of Food Science, Sichuan Agricultural University, No. 46, Xinkang Road, Yucheng District, Yaan 625014, China

**Keywords:** extrusion, temperature, soybean meal protein, structure, functional characteristics

## Abstract

Soybean meal protein, a byproduct of soybean processing, has limited functional properties such as emulsifying performance, which restricts its application in foods. Given that high-temperature extrusion tends to cause excessive denaturation and irreversible aggregation, this study aimed to investigate the effects of relatively low extrusion temperatures (85–105 °C) on the structural and functional properties of soybean meal protein. The results showed that extrusion altered the molecular structure and functional characteristics of the protein. With increasing extrusion temperature, the β-sheet content increased while the α-helix content decreased in the secondary structure, and tertiary structural rearrangements occurred, with hydrophobic groups being exposed and subsequently buried. At 95 °C, the protein formed a relatively porous and loose microstructure and exhibited the strongest surface hydrophobicity, water-holding capacity, oil-holding capacity, and emulsifying properties; at 100 °C and above, excessive aggregation occurred, pore structure collapsed, and functional properties declined. Meanwhile, extrusion generally reduced protein solubility. Therefore, 95 °C is identified as the optimal extrusion temperature under the conditions of this study. In addition, this study reveals the correlation between structural reconstruction and functional changes of soybean meal protein, providing a theoretical basis for its high-value utilization and application in the food industry.

## 1. Introduction

Soybean meal, a primary by-product of soybean oil production derived from either mechanical pressing or solvent extraction, contains 40–50% protein. This protein content is significantly higher compared to that found in corn, wheat, and other cereal grains. Soybean meal stands out as a high-quality plant-based protein resource. Its amino acid composition closely matches the nutritional requirements of livestock [1]. Currently, most soybean meal is used in livestock feed [2], with the remainder discarded. This represents an underutilization of its protein-rich composition. As a primary plant-based protein source, soybean meal has great potential to enhance its value further. Developing nutrient-dense and low-fat food products can enhance its functional value [3] and achieve sustainable reuse of agricultural by-products. However, native soybean meal protein has limited food applications. This is because its functional properties, such as emulsification [4], are restricted by its molecular conformation and aggregation state [5,6]. Therefore, modulation of the protein structure is one of the key approaches to improving its functional properties.

Extrusion processing, an efficient thermomechanical modification method, enhances protein functional properties by inducing structural reconfiguration through precise control of temperature, pressure, and shear forces [7,8]. Studies indicate that temperature critically governs protein denaturation and aggregation during the extrusion process [8,9]. Current methodologies predominantly employ broad temperature ranges (110–190 °C [10,11]), which pose a risk of excessive denaturation or irreversible aggregation, thereby constraining the functional potential of soybean meal proteins. Elucidating moderate-to-low-temperature extrusion’s effects on soybean meal protein structure and functionality is key to controlling its dissociation–aggregation balance for food applications. Research on the modulation of low-temperature extrusion remains limited, and there is a particular lack of systematic understanding regarding the transition boundary between moderate unfolding that favors functional improvement and excessive aggregation that leads to functional deterioration.

Therefore, the objectives of this study are not only to determine the optimal extrusion temperature but, more importantly, to elucidate the temperature-driven structural evolution pattern of dissociation–reaggregation of soybean meal protein within the narrow yet industrially feasible temperature range of 85–105 °C and to reveal its intrinsic correlations with water-holding capacity, oil-holding capacity, emulsifying properties, and foaming properties. By employing electrophoretic, spectroscopic, and microscopic analytical techniques, this study aims to define the temperature threshold at which functional improvement of the protein transitions into functional deterioration, thereby providing a mechanistic basis and processing rationale for the high-value food utilization of low-energy, low-temperature extrusion-modified soybean meal protein.

## 2. Materials and Methods

### 2.1. Materials

Soybean meal (46.63% crude protein, 10.85% moisture, 1.21% fat, 6.3% ash) was purchased from the agricultural market in Yucheng District, Ya’an City. Coomassie Brilliant Blue R250 and bromophenol blue were products of Sigma-Aldrich (St. Louis, MO, USA). Analytically pure reagents, including 8–anilino–1–naphthalenesulfonic acid (ANS), were procured from Yuanye Bio-Tech Co., Ltd. (Shanghai, China).

### 2.2. Extrusion Puffing

The moisture content was adjusted to 34% (wet basis) based on initial values. The amount of water added was calculated to achieve the target moisture content, taking into account the initial moisture of the raw material. The hydrated material was placed in polyethylene bags, hermetically closed, and left to equilibrate under ambient conditions for 24 h before extrusion. Extrusion was performed using a twin-screw extruder (model CLEXTRAL Ev025, CLEXTRAI Group, Firminy, France) fitted with a die containing a single 5 mm circular orifice. The screw configuration consisted of co-rotating, fully intermeshing elements, including conveying screws and kneading blocks, arranged in a standard food-processing assembly. The extruder was operated at a screw speed of 200 rpm and a feed rate of 2.5 kg/h. The specific mechanical energy (SME) was not calculated in this study, as the extruder was not equipped with an in-line torque sensor to record the actual mechanical energy input during processing. This absence of detailed screw configuration and SME data constitutes a limitation of the present study regarding the characterization of thermomechanical inputs. Barrel temperatures were set as follows: zone 1 was not actively heated (approximately 25 °C); zones 2 and 3 were set to five different temperature combinations in five separate runs—namely, (65/85 °C), (70/90 °C), (75/95 °C), (80/100 °C), and (85/105 °C). Post-extrusion, the expanded soybean meal samples were dried at 40 °C, mechanically pulverized, and sieved through an 80-mesh screen. The processed samples were stored in airtight bags at −20 °C for subsequent analyses. Untreated soybean meal served as the control group.

In this study, the extrusion temperature range of 85–105 °C was selected to establish a moderate-to-low-temperature extrusion window aimed at functional modification rather than high-temperature texturization processing conditions. This temperature range is sufficient, on the one hand, to induce partial denaturation and conformational rearrangement of soybean meal protein and, on the other hand, to avoid, to some extent, the severe irreversible aggregation caused by high-temperature extrusion above 110 °C. Therefore, this temperature window facilitates the identification of the transition boundary from moderate unfolding to excessive aggregation of the protein and provides a valuable reference for the industrial pretreatment of soybean meal protein targeting improved water-holding, oil-holding, and interfacial properties.

### 2.3. Protein Extraction

Protein extraction from extruded soybean meal was performed via alkaline extraction and isoelectric precipitation [12]. Briefly, soybean meal powder was combined with ultrapure water (1:25 *w*/*v*), and the mixture was brought to pH 8.5 with 1 M NaOH. The suspension was then placed in a constant-temperature water bath (model DKZ–2, Yiheng, Shanghai, China) with continuous shaking (1200 rpm) for 6 h. Subsequently, the mixture was centrifuged at 1550× *g* for 20 min at 4 °C using a refrigerated centrifuge (model TGL–20M, Luxiangyi, Shanghai, China) equipped with a No. 6 angle rotor (4 × 100 mL, maximum 13,000× *g* at 12,000 rpm). The supernatant was collected, adjusted to pH 4.5 with 1 M HCl, and incubated at 4 °C for 12 h to facilitate complete protein precipitation. After a second centrifugation (1550× *g*, 20 min, 4 °C) using the same centrifuge and rotor, the pellet was resuspended in five volumes of distilled water, neutralized to pH 7.0, and lyophilized for 24 h using a Shanghai Zhengqiao ZQ–FD–1A–50 freeze dryer (Shanghai Zhengqiao Scientific Instruments Co., Ltd., Shanghai, China). The lyophilized protein samples were stored at −20 °C until further analysis.

### 2.4. Sodium Dodecyl Sulfate–Polyacrylamide Gel Electrophoresis (SDS–PAGE)

Briefly, each sample was precisely weighed into centrifuge tubes, and sample buffer was added to give a protein concentration of 2 mg/mL (final). The mixtures were boiled at 100 °C for 3 min, then centrifuged at 14,800× *g* (equivalent to 12,000 rpm) for 1 min using a TG16G centrifuge (Kaite, Yancheng, China) with a No. 6 angle rotor (12 × 10 mL, maximum 14,800× *g* at 12,000 rpm) to remove insoluble debris. For electrophoresis, a 10% resolving gel and a 5% stacking gel were used, followed by fixation, staining, and destaining of the gel slabs.

### 2.5. Particle Size and Zeta Potential

A Malvern Zetasizer Nano ZS90 (Malvern Instruments Ltd., Worcestershire, UK) was used to measure the particle size and zeta potential of the samples. Protein samples were dissolved in deionized water at 10 g/L, then vortex-mixed for 30 min to ensure complete dissolution. After centrifugation at 872× *g* for 15 min at 25 °C using a TG16G centrifuge (Kaite, Yancheng, China) with a No. 9 rotor (4 × 100 mL, maximum 9690× *g* at 10,000 rpm), the supernatant was carefully taken, diluted 100-fold with deionized water to a final concentration of approximately 0.1 g/L, then used for measurements. The following instrument settings were applied: refractive index of 1.45, temperature of 25 °C, and equilibration time of 120 s.

### 2.6. Fourier-Transformed Infrared Spectroscopy (FTIR)

A Thermo Fisher Scientific Nicolet iS10 spectrometer (Thermo Fisher Scientific, Waltham, MA, USA)was used to record FTIR spectra for secondary structural analysis of the samples. The powdered material was thoroughly mixed with potassium bromide (KBr) at a mass ratio of 1:70, then pulverized evenly in an agate mortar. The mixture was pressed into transparent pellets using a hydraulic press, and spectra were collected over the range of 4000–400 cm^−1^ at a resolution of 4 cm^−1^ (32 scans). PeakFit 4.12 software was employed for spectral deconvolution.

### 2.7. Fluorescence Spectrum

The protein sample was adjusted to 1 mg/mL using 0.1 mol/L PBS (pH 7.0). Fluorescence spectra were recorded on a Thermo Fisher Scientific Lumina fluorescence spectrophotometer with the following settings: excitation at 290 nm, emission from 300 to 500 nm, 5 nm slits for both excitation and emission, and a photomultiplier tube (PMT) voltage of 500 V. The scan speed was set to 1200 nm/min, and the spectra were recorded at 25 °C using a 1 cm quartz cuvette. Triplicate measurements were averaged to generate the final spectrum.

### 2.8. Surface Hydrophobicity (H_0_)

A protein solution with a concentration of 0.2 mg/mL was prepared using ultrapure water. An aliquot of 4 mL of this solution was mixed with 20 μL of 8 mM ANS, vortexed, and incubated in the dark for 15 min. The fluorescence intensity was subsequently measured using a fluorescence spectrophotometer, with the maximum fluorescence intensity recorded as an indicator of protein surface hydrophobicity. The instrument parameters were set as follows: excitation wavelength of 390 nm, emission wavelength range of 400–600 nm, slit width of 5 nm, and photomultiplier tube (PMT) voltage of 400 mV. The scan speed was 1200 nm/min, and measurements were performed at 25 °C using a 1 cm quartz cuvette. Three independent replicates were measured for each sample.

### 2.9. Scanning Electron Microscope (SEM)

The sample was mounted on an SEM stub using conductive carbon tape, sputter-coated with a gold–palladium alloy, and loaded into a ZEISS Sigma 360 (Carl Zeiss AG, Oberkochen, Germany) scanning electron microscope. Imaging was conducted under optimized magnification and field-of-view settings to acquire high-resolution micrographs.

### 2.10. Protein Solubility

The protein sample was dispersed at a concentration of 1.00 mg/mL, then centrifuged at 1550× *g* for 30 min at 4 °C using the same refrigerated centrifuge and rotor (Luxiangyi TGL–20M, No. 6 angle rotor, 4 × 100 mL) as described in Section 2.3. The resulting clear supernatant was collected. Protein solubility was determined by the Bradford method using Coomassie Brilliant Blue G–250 reagent (Sigma-Aldrich (St. Louis, MO, USA)). A standard curve was generated using bovine serum albumin (BSA) by plotting absorbance against protein concentration. Solubility was expressed as a percentage of the soluble fraction relative to the total protein content.

### 2.11. Water- and Oil-Holding Capacity

Water-holding and oil-holding capacities were measured according to the method of Zhao [13] with slight adjustments. Pre-weighed 50 mL centrifuge tubes were used, and each tube received 0.5 g of protein sample, together with 5 mL of deionized water (or soybean oil). After vigorous vortex mixing, the suspension was allowed to stand for 30 min, then centrifuged at 872× *g* for 20 min using the same TG16G centrifuge and a No. 9 angle rotor as described in Section 2.5. The supernatant was removed by decantation, and the tube containing the residual protein was weighed. The following formula was used for calculation:

(1) $$\mathrm{Water}/\mathrm{oil}\;\mathrm{holding}\;\mathrm{capacity}\;(g/g)=\frac{M_{2}-M_{1}}{M_{0}}$$

In the formula, M_0_ is the mass of the protein sample (g), M_1_ is the mass of the centrifuge tube and protein (g), and M_2_ is the mass of the centrifuge tube and protein after water (oil) absorption (g).

### 2.12. Emulsifying Activity (EAI) and Stability (ESI) Indices

The characterization of protein-emulsifying properties was performed with reference to the method of Hu [14]. A 1 mg/mL protein solution (9 mL) was mixed with 1 mL of soybean oil. The mixture was homogenized using a Changzhou Yuexin RCD–1 A high-speed homogenizer (Changzhou Yuexin, Changzhou, China) at 12,000 rpm for 2 min to form an emulsion. At 0 min and 10 min after homogenization, 100 μL of the liquid was taken from the bottom of the beaker and added to 10 mL of a 0.1% (*w*/*v*) SDS solution. After mixing well, the SDS solution was used as a blank, and the absorbance of the diluted emulsion was measured at 500 nm. The emulsifying activity index (EAI) and emulsifying stability index (ESI) were calculated according to the following formulas:

(2) $$\mathrm{EAI}\;(m^{2}/g)=\frac{2\times T\times N\times A_{0}}{10,000\times C\times \Phi }$$

(3) $$\mathrm{ESI}\;(\min)=\frac{A_{0}}{A_{0}-A_{10}}\times \Delta T$$

In the formulas, T is the turbidity (2.303), N is the dilution factor, A_0_ represents the absorbance of the emulsion immediately after homogenization (0 min), C is the protein concentration (g/mL), Φ is the volume fraction of oil in the emulsion, A_10_ is the absorbance measured at 10 min after homogenization, and ∆T = 10 min.

### 2.13. Foaming Properties and Foam Stability

The measurement of the protein foaming properties was performed with reference to the methods of Wang [15], with slight adjustments. A 5.00 mg/mL protein solution was prepared, then homogenized at room temperature using a Changzhou Yuexin RCD–1 A high-speed homogenizer at 12,000 rpm for 2 min. The generated foam was immediately poured into a graduated cylinder. The volume of the solution before homogenization (v), the volume of the foam at the end of homogenization (v_0_), and the volume of the foam 10 min after homogenization (v_1_) were recorded. Foaming capacity (FC) and foaming stability (FS) were then determined using the equations below:

(4) $$\mathrm{FC}\;(\%)=\frac{V_{0}}{V}\times 100$$

(5) $$\mathrm{FS}\;(\%)=\frac{V_{1}}{V}\times 100$$

### 2.14. Statistical Analysis

IBM SPSS Statistics (version 27.0) was used for data analysis, and graphs were prepared with Origin Pro 2021. Each experiment was performed three times to ensure reproducibility. Data were expressed as mean ± SD, and differences were considered significant at *p* < 0.05.

## 3. Results and Discussion

### 3.1. SDS–PAGE

**Figure 1:**
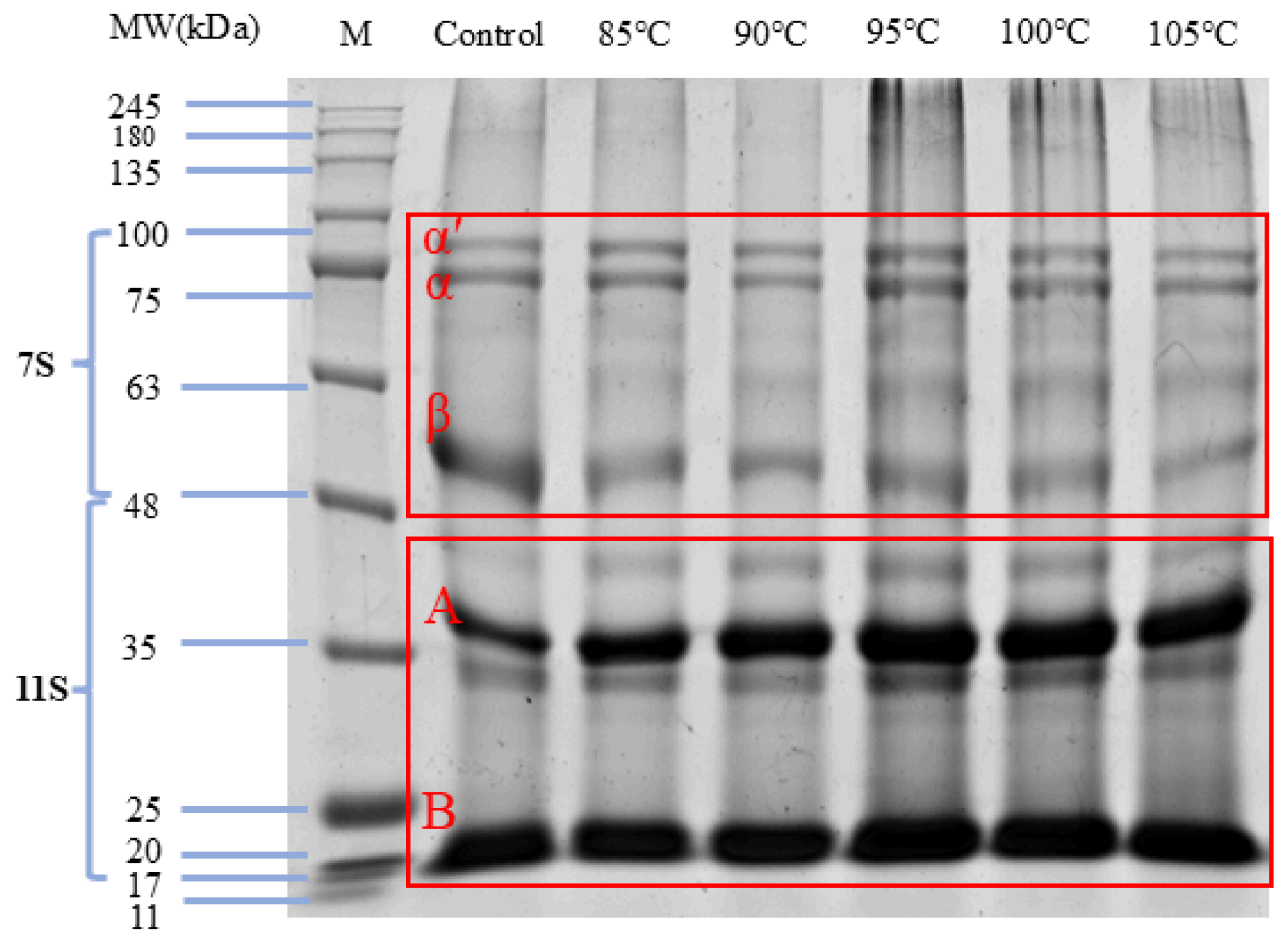
SDS–PAGE spectra of soybean meal protein after expansion at different extrusion temperatures. Lanes: M, protein molecular weight marker; Control, untreated soybean meal protein; 85–105 °C, extruded samples at the indicated temperatures. The bands corresponding to β-conglycinin (7S) subunits (α′, α, and β) and glycinin (11S) acidic (A) and basic (B) chains are labeled.

Figure 1 presents the molecular weight distribution of soybean meal protein under different extrusion temperatures. The main protein components in soybean meal are β-conglycinin (7S) and glycinin (11S). After extrusion, the 7S subunits (α, α’, and β) and 11S basic polypeptides (acidic/basic chains) showed clear changes in their molecular structure (Figure 1). Extruded samples exhibited broader protein-band distributions versus the control. The analysis revealed that band intensity exhibited an initial reduction phase, followed by progressive intensification at elevated temperatures. Notable polymer accumulation at sample loading positions was observed in protein lanes processed at 95 °C, 100 °C, and 105 °C, suggesting temperature-dependent protein aggregation [16]. In particular, glycinin (11S) is more susceptible to heat-induced aggregation than β-conglycinin (7S), likely through sulfhydryl-mediated crosslinking [17]. Mozafarpour et al. [11] demonstrated that at extrusion temperatures of 110 °C, 130 °C, and 160 °C, the 7S and 11S subunits of soybean meal protein exhibited complete band disappearance on SDS–PAGE, indicative of high-molecular-weight polymer formation during extrusion. These aggregates were unable to migrate through the separation gel matrix due to their size [18]. This study demonstrated that extrusion at 85–105 °C induced structural modifications in soybean meal protein. At lower temperatures (85–95 °C), protein dissociation occurred. Increasing extrusion temperatures (95–105 °C) enhanced dissociation, as evidenced by intensification of low-molecular-weight bands on SDS–PAGE, alongside partial re-aggregation and cross-linking of dissociated subunits into larger macromolecular aggregates. These findings align with prior reports on thermally driven protein structural transitions [19].

### 3.2. Particle Size

**Figure 2:**
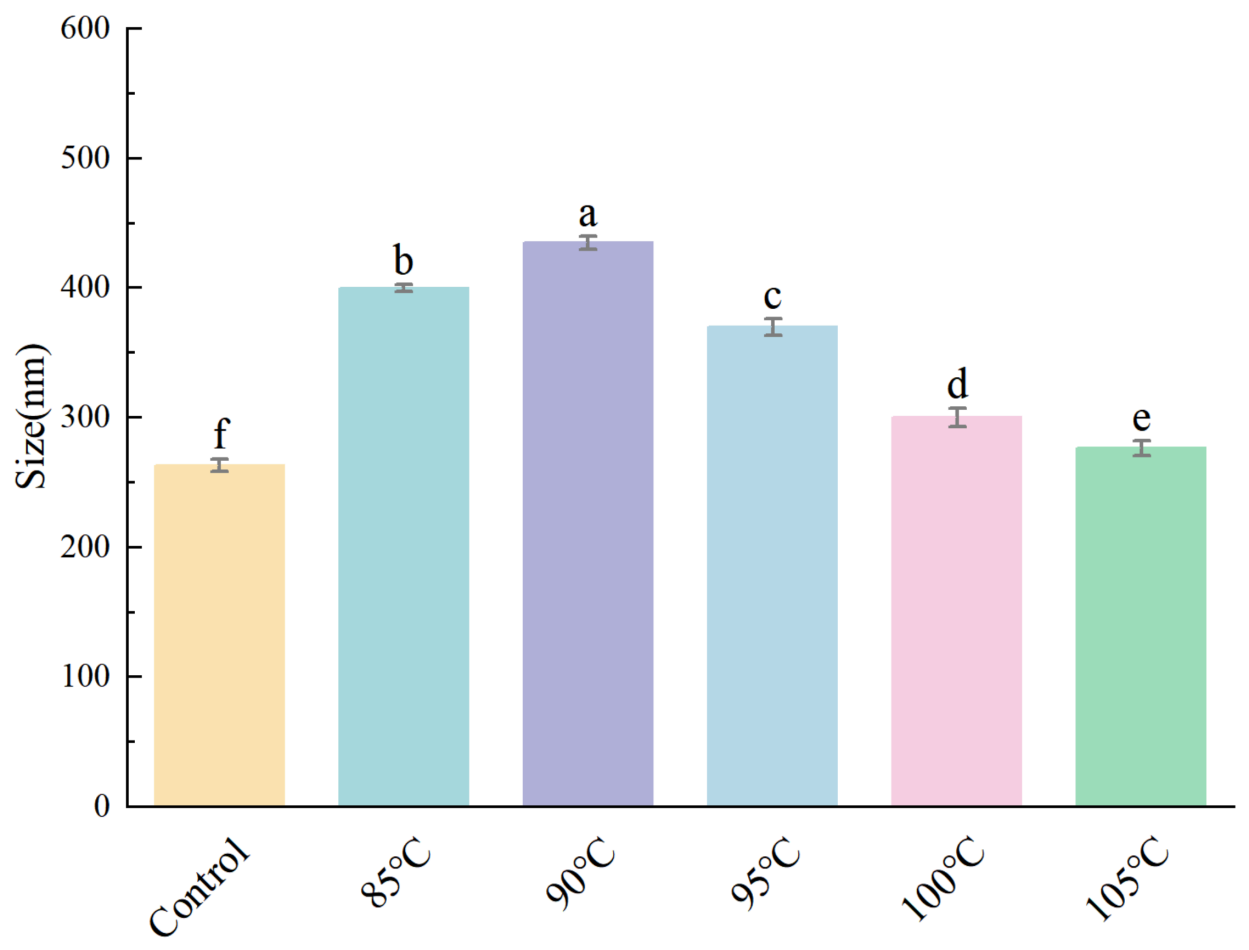
Particle size of soybean meal protein after puffing at different extrusion temperatures. Different letters indicate significant differences (*p* < 0.05).

Protein particle-size distribution serves as an indicator of spatial aggregation patterns, directly influencing functional properties. Figure 2 reveals significant particle-size enlargement in soybean meal protein following extrusion processing, suggesting structural aggregation or volumetric expansion induced by thermo-mechanical treatment. Increasing extrusion temperature caused the particle size of soybean meal protein to exhibit an initial increase followed by a decrease, indicating statistically significant alterations in particle dimensions occurred as the barrel temperature progressively rose. Moderate heat treatment promotes protein unfolding, which relaxes the compactly folded structure and enhances intermolecular encounters, consequently increasing particle size [20,21]. Upon further temperature increase, some large aggregates may be broken down by shear effects or rearrange into tighter conformations, causing the particle size to drop [22].

### 3.3. Zeta Potential

**Figure 3:**
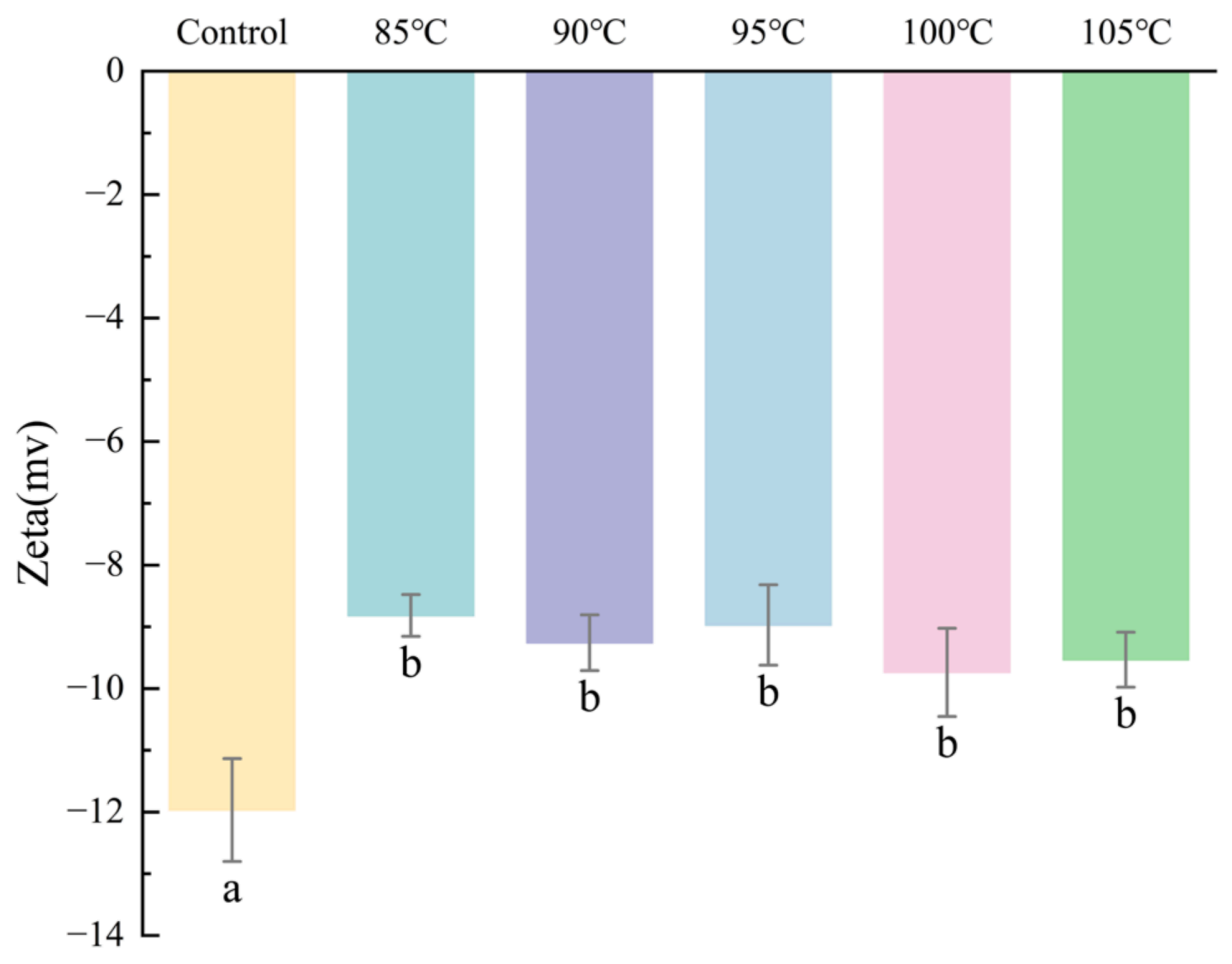
Zeta potential of soybean meal protein after puffing at different extrusion temperatures. Different letters indicate significant differences (*p* < 0.05).

Zeta potential is the electric potential at the shear plane of charged particles in solution; it describes the electrostatic interactions between particles and correlates with the distribution of surface charges on suspended particles. Particle surface charge affects both their own stability and their interactions with other particles. The higher the absolute value of zeta potential, the stronger the electrostatic repulsion among protein molecules, which improves the dispersion stability in solution systems [23]. The effective surface charge of protein molecules determines their dispersion and aggregation in solution. Figure 3 illustrates the zeta-potential profiles of soybean meal protein following extrusion at various temperatures. Figure 3 clearly shows a decrease in the absolute zeta potential of extruded soybean meal protein compared to the control. While insignificant differences were observed among different extrusion temperatures, this reduction is likely attributable to the barrel temperatures exceeding the protein’s denaturation threshold, promoting aggregate formation. This reduction in surface charge is associated with extrusion-induced structural changes.

### 3.4. FTIR

**Figure 4:**
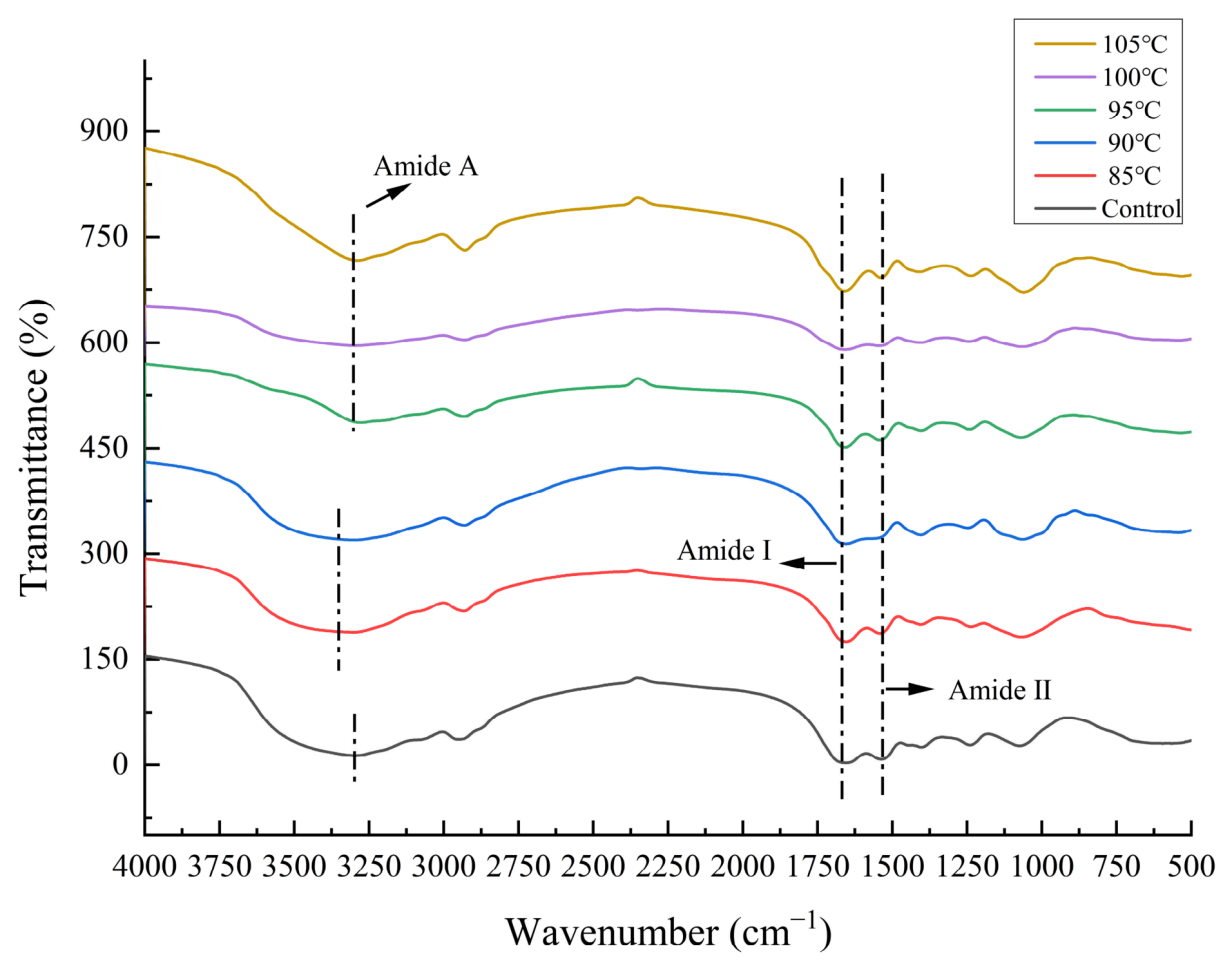
FTIR spectra of soybean meal protein after puffing at different extrusion temperatures.

FTIR spectroscopy was employed to investigate molecular structural modifications through functional-group vibrational analysis. Figure 4 reveals progressive modifications in absorption peaks between 3600 cm^−1^ and 3100 cm^−1^ with increasing extrusion temperatures, a spectral region characteristic of O–H and N–H stretching vibrations. This reduction may be attributed to extrusion-induced alterations in intermolecular or intramolecular hydrogen bonding [24]. At extrusion temperatures of 85 °C and 90 °C, the Amide A band exhibited a significant blue shift. This is likely due to protein unfolding induced by extrusion, which exposes N–H groups originally involved in hydrogen bonding, leaving them in a free state and thereby reducing hydrogen-bond formation. The concomitant decrease in absorption intensity may reflect changes in the hydrogen bonding environment of N–H groups, which could restrict their effective vibration. Conversely, at extrusion temperatures of 95 °C, 100 °C, and 105 °C, the characteristic peak exhibited a red shift. This was likely due to denaturation and aggregation of some proteins when exposed to high temperature, pressure, and shear simultaneously, which encourages the creation of new hydrogen bonds. The concomitant decrease in overall peak intensity may result from structural disorganization.

The secondary structure of proteins, made up of α-helices, β-sheets, β-turns, and random coils, arises from hydrogen-bond-mediated polypeptide chain folding and serves as the structural foundation for three-dimensional conformation [25]. The amide I band (1600 cm^−1^ to 1700 cm^−1^) was deconvoluted using PeakFit 4.12 (Table 1), yielding the relative contents of protein secondary structure components: α-helix (1650–1660 cm^−1^), β-sheet (1610–1640 cm^−1^), β-turn (1660–1670 cm^−1^), and random coil (1640–1650 cm^−1^) [26].

As shown in Table 1, extrusion processing induced significant alterations in secondary protein structure relative to untreated controls. The substantial increase in β-sheet content observed in thermally modified samples likely reflects partial denaturation and structural disordering, which may be associated with changes in surface hydrophobicity. This structural reorganization was accompanied by improved emulsification capacity in extruded soybean meal protein, consistent with previous findings by Chen et al. [27]. Increasing extrusion temperature significantly decreased the α–helix content in the secondary structure of soybean meal protein, likely due to enhanced protein unfolding under thermal processing. Concurrently, the random coil content increased, indicating more flexible regions formed at elevated temperatures [28,29]. Greater conformational flexibility typically enables protein molecules to diffuse and rearrange more rapidly at oil–water and air–water interfaces, thereby offering a structural foundation for enhanced emulsifying and foaming performance [30,31].

### 3.5. Fluorescence Spectrum

**Figure 5:**
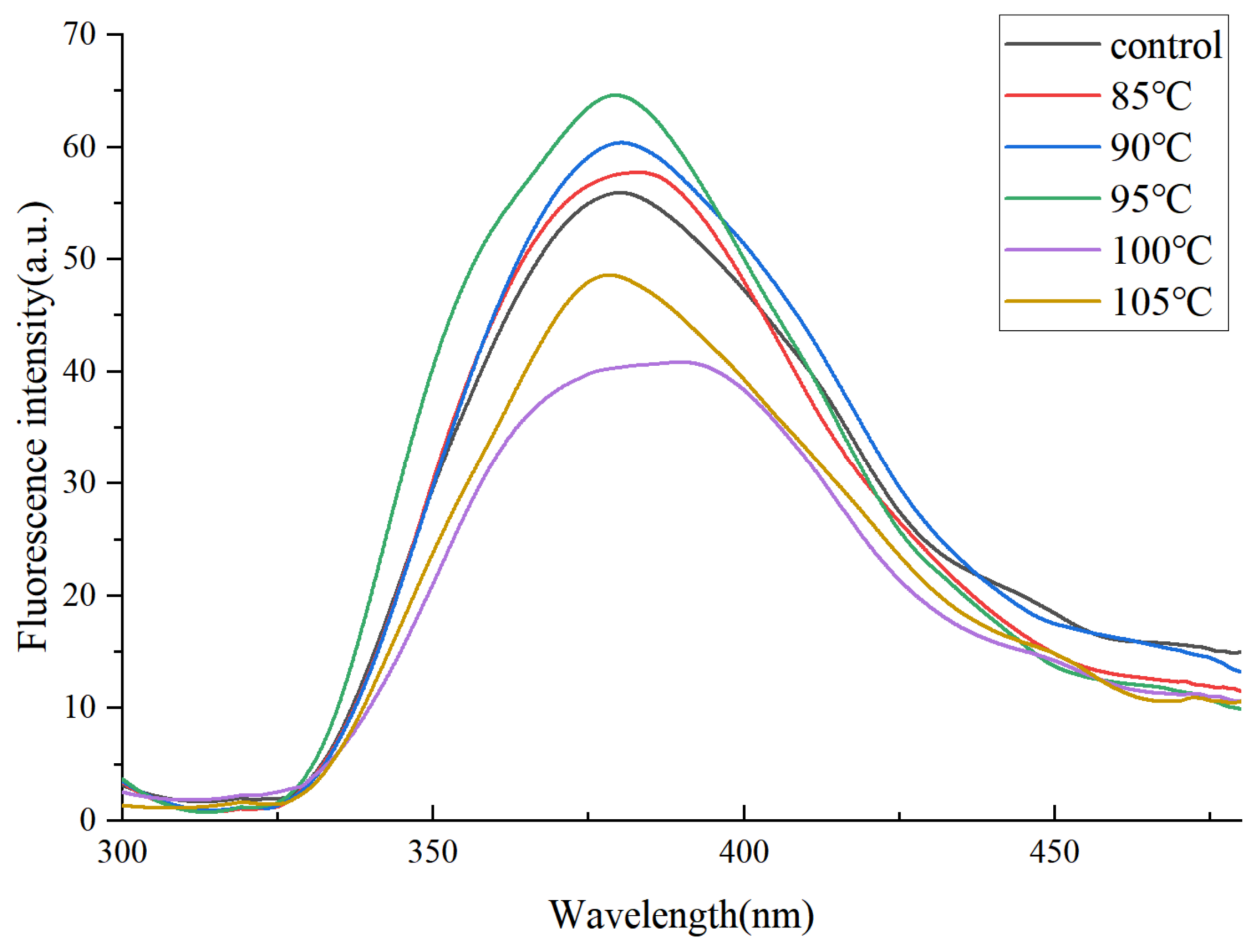
Endogenous fluorescence spectra of soybean meal protein after expansion at different extrusion temperatures.

Intrinsic fluorescence spectroscopy probes tertiary protein structure, which arises specifically from the aromatic residues of tyrosine (Tyr) and tryptophan (Trp). In soybean meal protein, the indole group of tryptophan (Trp) serves as the primary fluorophore. Owing to its high sensitivity to solvent polarity, changes in protein internal microenvironmental polarity are reflected in alterations of the fluorescence spectrum. Figure 5 shows that, relative to the control, the fluorescence intensity of the extruded protein first increased, then decreased as the extrusion temperature rose. At 85 °C, 90 °C, and 95 °C, intensity generally increased, indicating that progressive protein unfolding with rising extrusion temperatures led to greater exposure of fluorophores. Contrastingly, at extrusion temperatures of 100 °C and 105 °C, fluorescence intensity decreased due to quenching. This likely resulted from previously exposed hydrophobic groups reassociating or aggregating into more stable structures under elevated temperatures. Compared with 100 °C, the slight intensity increase at 105 °C may be related to structural rearrangements affecting the local environment of tryptophan residues [32]. Greater conformational flexibility typically facilitates faster diffusion and rearrangement of protein molecules at both oil–water and air–water interfaces, which, in turn, provides a structural basis for improved emulsifying and foaming performance [33].

### 3.6. Surface Hydrophobicity

**Figure 6:**
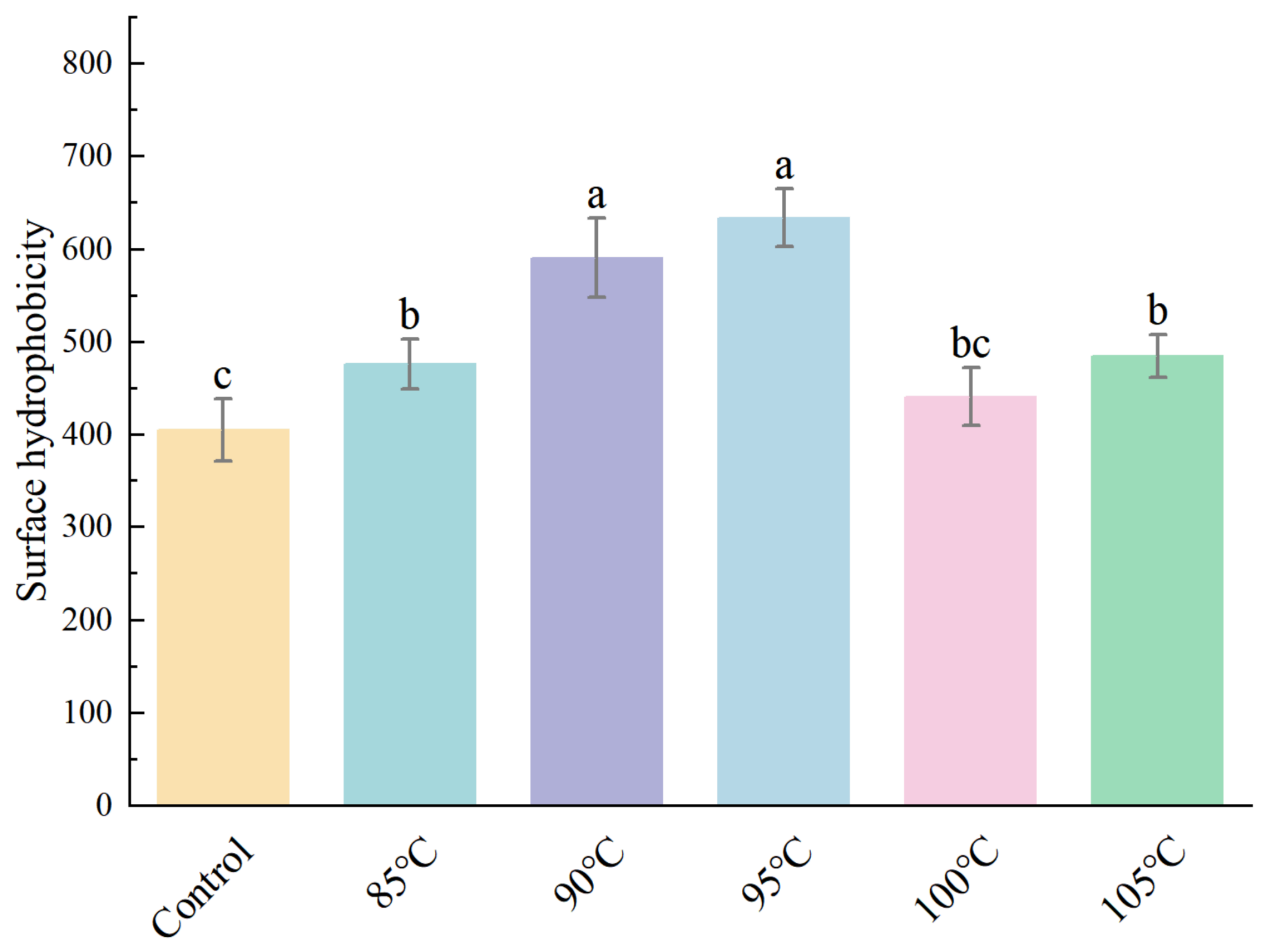
Surface hydrophobicity of soybean meal protein following extrusion at varying temperatures. Different letters indicate significant differences (*p* < 0.05).

Surface hydrophobicity (H_0_) of proteins, which reflects the exposure of hydrophobic residues, can markedly influence interfacial properties, including emulsification and foaming [34]. The surface hydrophobicity of a protein correlates positively with the amount of exposed hydrophobic moieties. Measuring surface hydrophobicity allows for estimation of these groups in aqueous solution [35], evaluation of tertiary structural changes [36], and prediction of functional properties such as solubility and emulsifying capacity [37,38]. As illustrated in Figure 6, the surface hydrophobicity of soybean meal protein initially increases with rising extrusion temperature, peaking at 95 °C before subsequently declining. This likely occurred because extrusion between 85 and 95 °C induces protein loosening, leading to greater exposure of hydrophobic groups. At 100 °C, surface hydrophobicity decreased significantly, presumably attributable to protein aggregation induced by the synergistic action of heat, pressure, and shear within the extruder, thereby burying surface-exposed hydrophobic groups [29]. Soybean meal protein extruded at 105 °C exhibited a slight increase in surface hydrophobicity. This may result from partial dissociation of protein aggregates or subunits at this temperature, enhancing ANS binding [39], consistent with fluorescence spectroscopy data. Moreover, it has been reported that enhanced surface hydrophobicity is typically associated with reduced α-helix content and that increased hydrophobicity often correlates with heightened intrinsic fluorescence intensity [40]; these observations were confirmed in Section 3.4 and Section 3.5.

### 3.7. SEM

**Figure 7:**
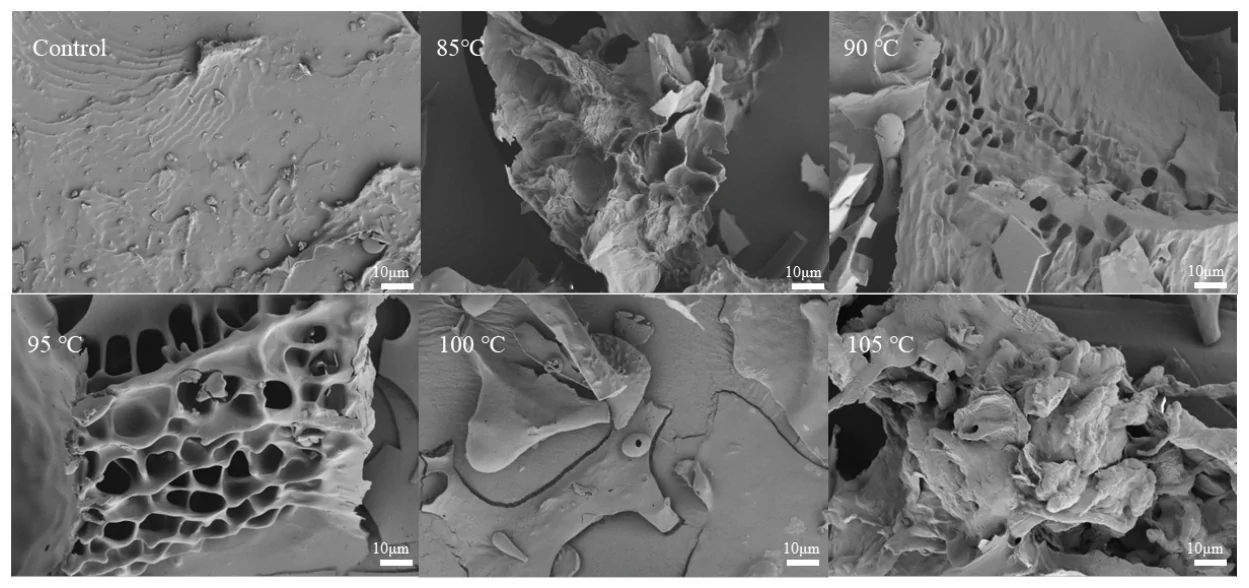
Scanning electron microscopy (SEM) images of soybean meal protein.

As shown in Figure 7, the surface of the control group exhibits a relatively smooth morphology, whereas the extrusion-puffed sample displays a notably rougher texture, with significantly higher surface roughness and the presence of porous structures. At an extrusion temperature of 95 °C, a continuous and maximally porous structure forms on the protein surface. This morphological change is likely due to the combined effects of high temperature, high pressure, and shear forces within the barrel, which induce expansion of the protein matrix. At 100 °C and 105 °C, the porous protein structure disintegrates, and the protein surface shows signs of fusion and collapse, suggesting that smaller protein entities associate into larger, denser aggregates. An appropriate porous structure facilitates an increase in specific surface area and interfacial contact sites, thereby improving its water-holding capacity, oil-holding capacity, and emulsifying performance [41].

### 3.8. Protein Solubility

**Figure 8:**
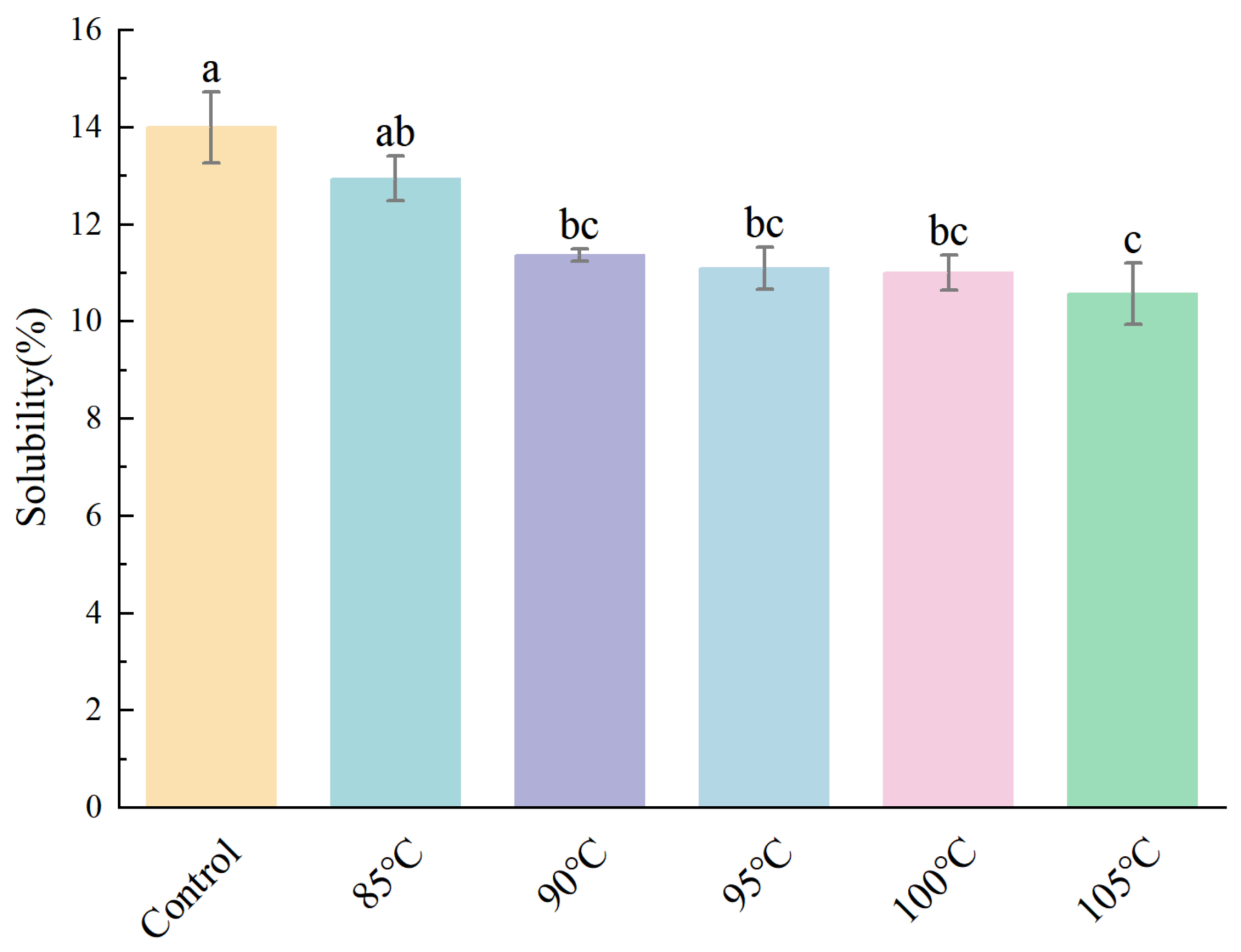
Solubility of soybean meal protein following extrusion at varying temperatures. Different letters indicate significant differences (*p* < 0.05).

Proteins exist as organic macromolecular compounds in a dispersed (colloidal) state in water. The extent of their aqueous dispersion defines protein solubility. Changes in protein solubility serve as an indicator of denaturation extent. Figure 8 displays the solubility values of soybean meal protein after extrusion at various temperatures. Extrusion caused a marked decrease in protein solubility (*p* < 0.05). Extrusion at 105 °C induced substantial protein denaturation, correlating with markedly reduced solubility. SEM characterization revealed expanded porous microstructures in proteins processed at 85 °C, 90 °C, and 95 °C, yet their solubility remained significantly below that of the untreated control. The decrease in solubility may be explained by extrusion-induced disruption of secondary bonds that maintain tertiary protein structure under combined high temperature, pressure, and shear forces, leading to protein unfolding and exposure of hydrophobic groups. These exposed hydrophobic groups can reassociate under the given temperature and moisture conditions, forming thermally induced insoluble aggregates [42]. In addition, the solubility of heat-denatured protein during puffing also depends on its molecular size [43]. The increased particle size observed in Figure 2 supports this association.

### 3.9. Water- and Oil-Holding Capacity

**Figure 9:**
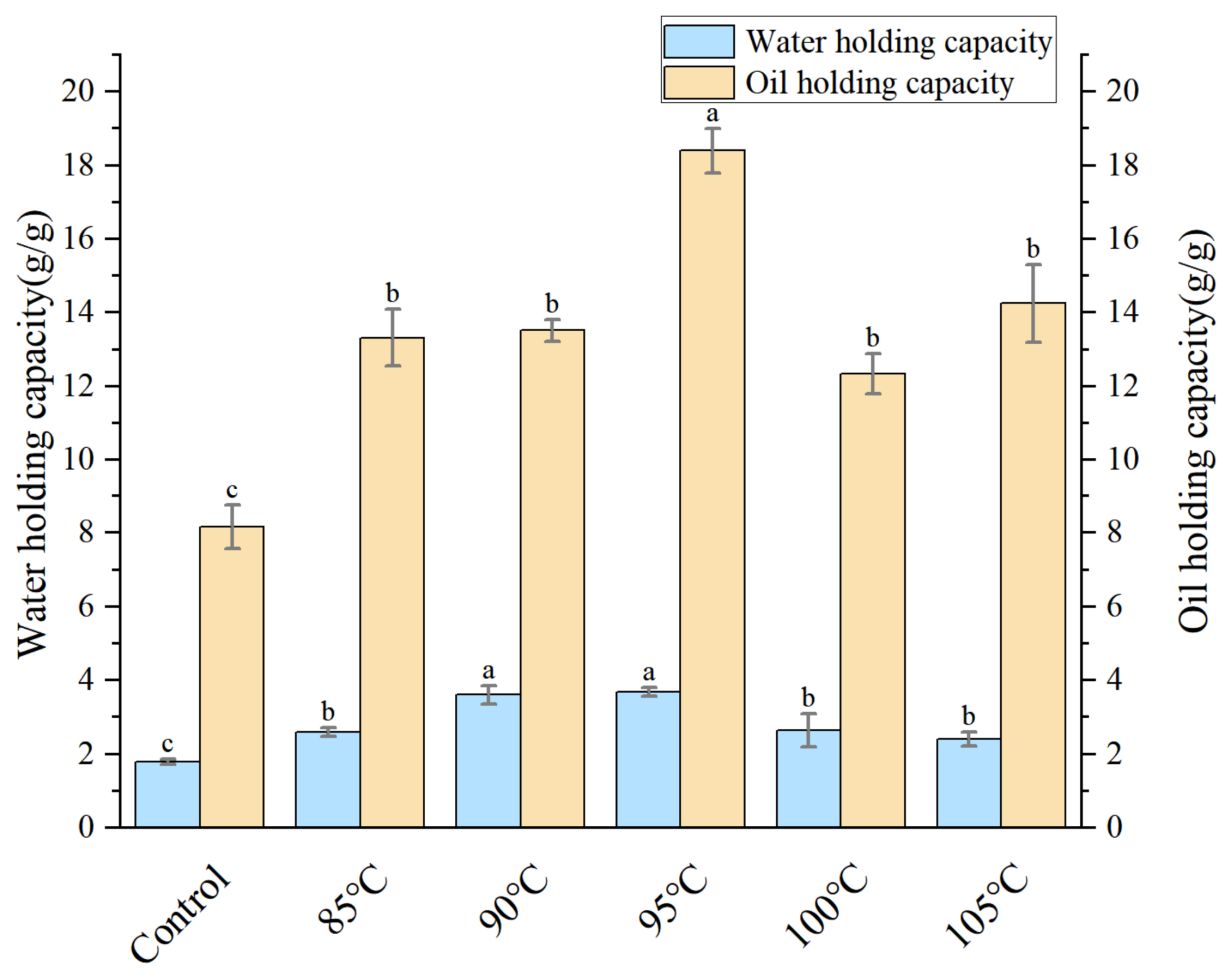
Water and oil retention of soybean meal protein after puffing at different extrusion temperatures. Different letters indicate significant differences (*p* < 0.05).

As essential functional characteristics of proteins, water-holding and oil-holding capacities are fundamentally attributed to physical entrapment mechanisms. A protein’s water- or oil-holding capacity is the maximum mass of water or oil retained per unit mass of dry protein upon direct interaction. According to Figure 9, the water-holding and oil-holding capacities of the puffing group showed a significant increase compared with those of the control group. As the extrusion temperature increased, the water- and oil-holding properties first increased, then decreased. The water-holding capacity was 1.78 ± 0.07 g/g in the control group, which increased to 3.68 ± 0.12 g/g during extrusion at 95 °C but decreased to 2.39 ± 0.20 g/g upon an increase in the extrusion temperature to 105 °C. The primary reason for this phenomenon is that low-temperature extrusion of soybean meal protein induces structural expansion and pore formation. The elevated temperature induces the shrinkage of surface pores initially present within the protein architecture, resulting in the creation of a more compact architecture. This phenomenon may be associated with structural rearrangements within the protein matrix [44], which aligns with prior structural protein findings. Considering the decrease in solubility after extrusion (Figure 8), it is suggested that these enhanced functional properties are not primarily dependent on the high solubility state of protein molecules but more likely originate from the porous structure resulting from moderate unfolding and the exposure of hydrophobic sites.

Protein pores expand progressively when extruded at 85 °C, 90 °C and 95 °C, enhancing water absorption. The increased contact area between the two components facilitates their interaction, thereby improving the water-holding capacity of soybean meal proteins. Proteins extruded at 100 °C and 105 °C demonstrated lower surface-pore density. The direct interaction between the protein and water is hindered, impairing its capacity to bind water and consequently reducing the water-holding ability of soybean meal protein [45].

The oil-holding capacity of a protein is influenced by multiple factors, including the number of hydrophobic groups present on its surface, as well as its structural porosity and morphology. Compared with the control group, the change trend of oil-holding capacity in the expansion group was largely consistent with that of water-holding capacity. The maximum oil-holding capacity (18.39 ± 0.6 g/g) was recorded at 95 °C, while the minimum (12.33 ± 0.55 g/g) occurred at 100 °C. At lower temperatures, the protein structure undergoes stretching under compression, leading to the exposure of non-polar groups and consequently enhancing the oil-holding capacity. At elevated temperatures, the binding interactions between protein molecules are enhanced, leading to aggregate formation. This reduces the proteins’ contact area with lipids and consequently decreases the oil-holding capacity [46].

### 3.10. Emulsifying Activity and Stability Indices

**Figure 10:**
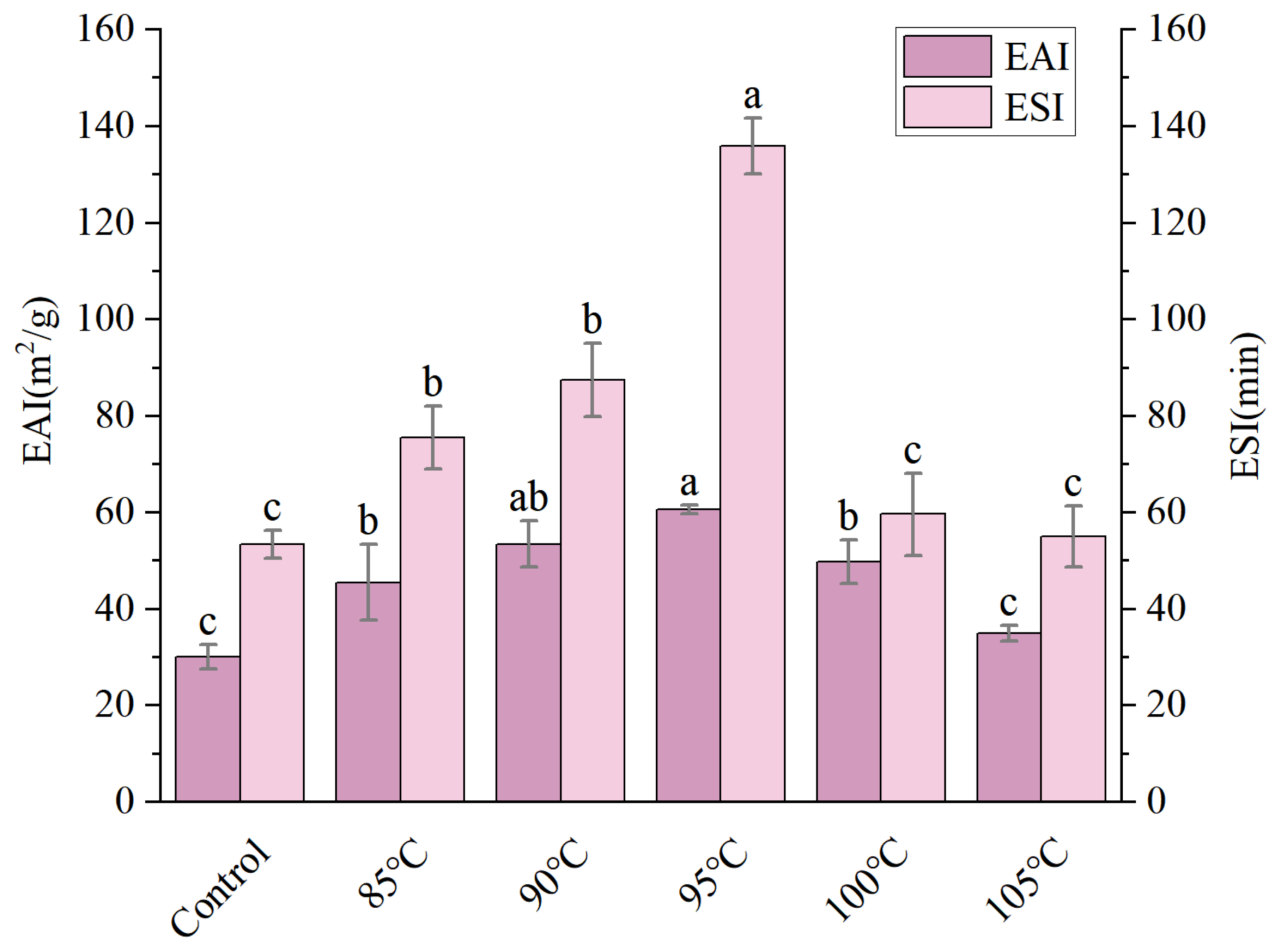
EAI and ESI of soybean meal protein after puffing at different extrusion temperatures. Different letters indicate significant differences (*p* < 0.05).

Emulsification capacity is a crucial functional property of proteins, encompassing two primary aspects: emulsifying ability and emulsion stability. The emulsifying property of proteins refers to their ability to stabilize the oil–water interface by forming an emulsion through adsorption onto the oil surface when a protein solution is mixed with oil. The emulsifying stability refers to the ability of an oil–water emulsion to resist destabilization under external environmental conditions. As illustrated in Figure 10, both EAI and ESI exhibit a variation pattern that initially increases, then decreases with rising extrusion temperature, peaking at 95 °C. This trend is consistent with the extrusion-induced changes in secondary protein structure (Section 3.4), where an increasingly disordered structure and flexibility may enhance protein adsorption at the oil–water interface, thereby improving emulsification properties [27,47]. In addition, the maximum surface hydrophobicity exhibited by soybean meal protein extruded at 95 °C was also favorable for its emulsifying performance. Despite the concomitant reduction in solubility, the greater exposure of hydrophobic domains likely improves the protein’s ability to adsorb at oil–water interfaces, thereby offsetting the solubility decrease—an observation that agrees with the report by Gharsallaoui and co-workers [48]. The relatively high extrusion temperature induces protein aggregation and may disrupt certain hydrophobic groups, thereby altering the arrangement of nonpolar and polar amino acid residues across the protein surface [49]. Extruded soybean meal protein showed enhanced emulsification stability, which may be related to the formation of a protective layer at the oil–water interface during homogenization [11,50].

### 3.11. Foaming Properties and Foam Stability

**Figure 11:**
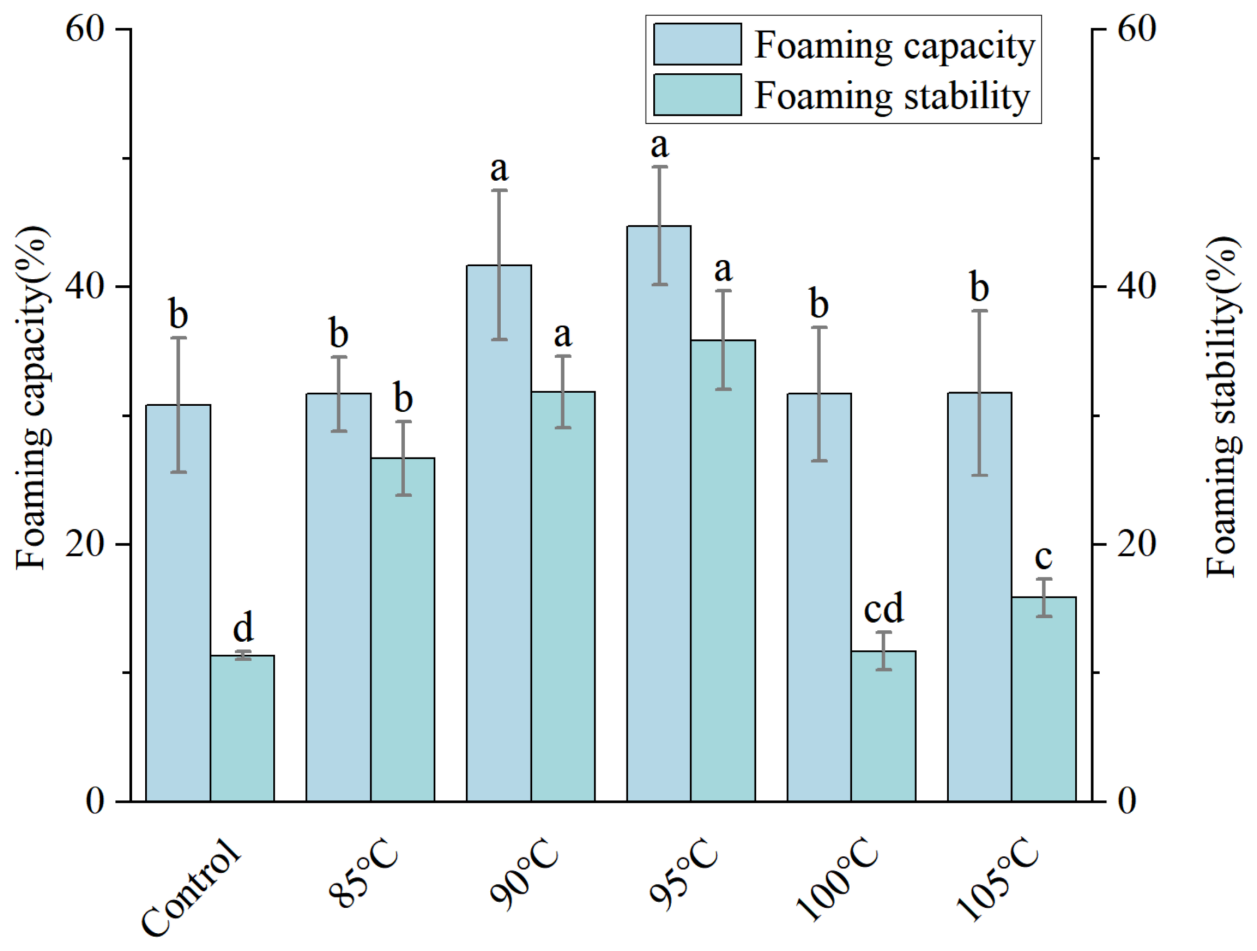
Foaming properties and foam stability of soybean meal protein following expansion at various extrusion temperatures. Different letters indicate significant differences (*p* < 0.05).

The foaming properties of proteins encompass both foaming capacity and foam stability. Foaming ability describes the volume of foam generated by proteins under shear conditions, while foam stability refers to the capacity of the produced foam to persist over time without collapsing [51]. Baier et al. [52] reported that the enhanced foam performance could potentially be attributed to the increase in H_0_. An increase in H_0_ can promote foam formation, thereby enhancing the protein’s foaming performance. Figure 11 reveals that as extrusion temperature rises, the foaming property and foam stability of soybean meal protein show a trend of first increasing, then decreasing, and this change is consistent with the change at 3.6 H_0_. Furthermore, the potential cause stems from changes in the protein’s higher-order structure under relatively low-temperature compression. This process leads to molecular stretching and structural reorganization, thereby enhancing both the foaming capacity and foam stability of the protein [53]. When the temperature is excessively high, protein molecules undergo coagulation, causing their molecular structure to become more compact. This structural change reduces the ability to stabilize foam, consequently impairing their foaming performance.

### 3.12. Extrusion Temperature-Mediated Structure–Function Mechanism of Soybean Meal Protein

Combining the results of SDS–PAGE, particle size, zeta potential, FTIR, intrinsic fluorescence, surface hydrophobicity, and SEM, it is evident that soybean meal protein undergoes a continuous structural evolution from “partial dissociation and unfolding” to “excessive reaggregation” during extrusion at 85–105 °C, and the changes in functional properties are essentially governed by the dynamic balance between protein unfolding and aggregation.

At lower extrusion temperatures (85–90 °C), the synergistic action of heat, pressure, and shear first disrupts some of the non-covalent interactions—including hydrogen bonds and hydrophobic interactions—that maintain the native globular structure, leading to a certain degree of dissociation and unfolding of the protein. This process is characterized by a decrease in α-helix content and increases in random-coil and β-sheet contents, along with enhanced intrinsic fluorescence, indicating the progressive exposure of aromatic residues and hydrophobic groups originally buried within the molecular interior. Moderate unfolding increases the conformational flexibility of the protein, which facilitates its interactions with the aqueous phase, oil phase, and interfaces, thereby initiating the improvement of relevant functional properties.

When the extrusion temperature is raised to 95 °C, the unfolding and aggregation of the protein reach a relatively favorable equilibrium. On one hand, surface hydrophobicity reaches a relatively high level, indicating that hydrophobic sites involved in lipid binding and interfacial adsorption are fully exposed; on the other hand, SEM observations reveal a relatively loose and porous microstructure at this temperature, which provides greater physical entrapment space for water and oil and increases the contact area with the external phase. Consequently, the sample extruded at 95 °C exhibits the highest water-holding capacity, oil-holding capacity, emulsifying properties, and foaming properties. Thus, 95 °C is not merely an empirical “optimal temperature” but represents a critical structural state in which soybean meal protein transitions from moderate unfolding that favors functional improvement to excessive aggregation that is detrimental.

When the extrusion temperature is further elevated to 100–105 °C, excessive heat input intensifies interactions among protein subunits, promoting the reaggregation of dissociated protein molecules into denser macromolecular aggregates. The accumulation of polymers at the wells in SDS-PAGE, the diminished fluorescence intensity, and the pore collapse and surface fusion observed in SEM all support this interpretation. At this stage, some of the previously exposed hydrophobic groups become partially re-buried within the aggregates, impairing the interfacial migration and rearrangement capacity of protein molecules, while the dense structure also weakens the entrapment of water and oil, ultimately resulting in decreased water-holding capacity, oil-holding capacity, emulsifying properties, and foaming properties.

In summary, the regulation of functional properties of soybean meal protein by extrusion temperature essentially arises from the sequential transition of the protein from a native compact structure to a moderately unfolded porous structure, then to an excessively aggregated dense structure.

## 4. Conclusions

This study investigated the effects of extrusion temperatures on the structural and functional properties of soybean meal protein. The results demonstrated that extrusion temperature critically governs protein molecular conformation and aggregation behavior, thereby modulating its functional performance. Increasing extrusion temperature progressively induced dissociation, reaggregation, and cross-linking of soybean meal protein under combined thermal, pressure, and shear stress. Overall, the soybean meal protein extruded at 95 °C achieves a favorable balance between molecular unfolding and aggregation, resulting in a structural state characterized by relatively high surface hydrophobicity; enhanced conformational flexibility; and a loose, porous morphology, all of which collectively contribute to improved water-holding, oil-holding, emulsifying, and foaming properties. From an application perspective, this type of extrusion-modified protein is more suitable as a food ingredient with water- and oil-holding and interface-stabilizing functions and can be potentially applied in minced meat products, plant-based emulsified systems, and low-fat formulated foods. Compared with high-temperature extrusion, the moderate-to-low-temperature extrusion strategy proposed in this study is expected to reduce the risk of excessive thermal aggregation while enabling targeted functional modification of soybean meal protein, thereby providing a processing reference for its transformation from a low-value feed byproduct to a high-value-added functional food ingredient.

## Figures and Tables

**Table 1:** Analysis of secondary structure content in soybean meal protein before and after expansion at different extrusion temperatures.

Sample	Secondary Structure Content (%)			
	α-Helix	β-Sheet	β-Turn	Random Coil
Control	19.87 ± 0.45 ^a^	33.33 ± 0.53 ^c^	30.52 ± 0.11 ^a^	16.28 ± 0.16 ^d^
85 °C	18.67 ± 0.34 ^b^	34.94 ± 0.45 ^ab^	29.47 ± 0.16 ^b^	16.92 ± 0.02 ^c^
90 °C	18.16 ± 0.08 ^c^	35.36 ± 0.35 ^a^	29.75 ± 0.65 ^b^	16.72 ± 0.33 ^c^
95 °C	18.24 ± 0.04 ^c^	34.67 ± 0.04 ^b^	29.65 ± 0.18 ^b^	17.44 ± 0.1 ^b^
100 °C	17.23 ± 0.24 ^c^	35.42 ± 0.25 ^a^	30.06 ± 0.69 ^ab^	17.29 ± 0.19 ^b^
105 °C	17.16 ± 0.15 ^c^	35.19 ± 0.05 ^a^	29.61 ± 0.16 ^b^	18.04 ± 0.03 ^a^

Values are mean ± SD (*n* = 3). Different letters in the same column indicate significant differences (*p* < 0.05).

## Data Availability

The raw data supporting the conclusions of this article will be made available by the authors on request due to ongoing research and data management policies; however, they can be obtained from the corresponding author upon reasonable request.
